# A new species of smooth skink (Squamata: Scincidae: *Scincella*) from Cambodia

**DOI:** 10.24272/j.issn.2095-8137.2018.008

**Published:** 2018-04-28

**Authors:** Thy Neang, Somaly Chan, Nikolay A. Poyarkov

**Affiliations:** 1 Wild Earth Allies, Sk. Phnom Penh Thmei, Kh. Sen Sok, Phnom Penh, Cambodia; 2 Ministry of Environment, Sk. Tonle Bassac, Kh. Chamkarmorn, Phnom Penh, Cambodia; 3 Department of Vertebrate Zoology, Biological Faculty, Lomonosov Moscow State University, Moscow 119234, Russia; 4 Joint Russian-Vietnamese Tropical Research and Technological Centre, Nghia Do, Cau Giay, Hanoi, Vietnam

**Keywords:** Mondulkiri, Keo Seima Wildlife Sanctuary, Taxonomy, mtDNA, *COI*, DNA barcoding, Phylogenetics

## Abstract

Based on morphological and genetic evidence we evaluated the taxonomic status of a newly discovered forest-dwelling population of skink (genus *Scincella*) from the Keo Seima Wildlife Sanctuary, Mondulkiri Province, Cambodia. From phylogenetic analysis of a 668-bp fragment of the mtDNA *COI* and diagnostic morphological characters we allocate the newly discovered population to the *Scincella reevesii–S. rufocaudata* species complex and describe it as *Scincella nigrofasciata* **sp. nov.** The new skink species can be distinguished from all other Southeast Asian congeners by the following combination of morphological characters: snout-vent length (SVL) 40.0–52.6 mm; relative tail length (TaL/SVL ratio) 1.25–1.94; prefrontals in broad contact; infralabials 6; primary temporals 2; relative forelimb length (FIL/SVL ratio) 0.20–0.22; relative hindlimb length (HIL/SVL ratio) 0.30–0.33; relative forearm length (FoL/SVL ratio) 0.14–0.16; adpressed forelimbs and hind limbs either overlapping (0.4–2.2 mm) or separated (1.9–2.3 mm); midbody scale rows 32–33, paravertebral scales 69–74, vertebral scales 65–69; dorsal scales between dorsolateral stripes 8; comparatively slender fingers and toes, subdigital lamellae under fourth toe 15–17; dark discontinuous regular dorsal stripes 5–7; distinct black dorsolateral stripes, narrowing to lateral sides and extending to 52%–86% of total tail length. We provide additional information on the holotype of *Scincella rufocaudata* (Darevsky & Nguyen, 1983), and provide evidence for the species status of *Scincella rupicola*. Our discovery brings the number of *Scincella* species in Cambodia to five and emphasizes the incompleteness of knowledge on the herpetofaunal diversity of this country.

## INTRODUCTION

The family Scincidae is one of the most globally diverse groups of lizards with 146 genera and about 1 650 species currently recognized worldwide (Uetz et al., 2018). Of these, the smooth skink genus *Scincella* Mittleman, 1950 currently contains 34 species with fragmented distribution, from the North American continent (five species) to Japan, Ryukyu Archipelago and Taiwan, China, Korean Peninsula, mainland China, and Southeast Asia (remaining species) (Ouboter, 1986; Uetz et al., 2018). *Scincella* species are characterized by their small size, elongated body, short limbs, relatively long tail, smooth subcycloid scales (most species), small oblong head with transparent disc in a movable lower eyelid, absence of supranasals, pentadactyl hindlimbs, one row of basal subdigital lamellae (most species), median preanals overlapping lateral ones, four or more scales bordering the parietals between the upper secondary temporals, and lower secondary temporal overlapping the upper one (diagnosis follows Greer & Shea, 2003; Lim, 1998; Nguyen et al., 2010a, 2010b, 2010c). Furthermore, the genus *Scincella* is differentiated from closely related *Sphenomorphus* Fitzinger by the presence of a transparent window in the lower eyelid as opposed to lower eyelid covered with polygonal scales in *Sphenomorphus* (Greer, 1974; Nguyen et al., 2010a).

The phylogenetic relationships of *Scincella* and many other Southeast Asian lygosomine skinks remain unresolved because they share many morphological similarities (e.g., Nguyen et al., 2010a, 2010b). Based on examination of museum specimens, Ouboter (1986) undertook a major revision of *Scincella* Mittleman, 1950, which resulted in numerous synonymies, some of which are discussed in the present paper (see Discussion). The morphological similarities and taxonomic uncertainty have hampered further progress in the systematics of smooth skinks, with only a few species described in the last 15 years, including three taxa discovered from Vietnam (Darevsky et al., 2004; Nguyen et al., 2010a, 2010b) and one from Mexico (García-Vázquez et al., 2010). In the present paper, we follow the taxonomy proposed by Darevsky (1990), who transferred *Sphenomorphus rufocaudatus* Darevsky & Nguyen, 1983 to the genus *Scincella* as *Scincella rufocaudata* (Darevsky & Nguyen, 1983) without providing any detailed information on this assignment. This taxonomy was accepted subsequently by Nguyen et al. (2011) and Neang & Poyarkov (2016). *Scincella rufocaudata* was reported from Cambodia by Stuart et al. (2006) and Stuart & Emmett (2006) based on specimens from the Mondulkiri Province and Cardamom Mountains of southwest Cambodia (see Discussion). Therefore, to date, the genus *Scincella* in Cambodia is represented by four species: that is, *S. melanosticta* (Boulenger), *S.* cf. *rufocaudata* (Darevsky & Nguyen), *S. reevesii* (Gray), and *S.* cf. *rupicola* (Smith) (Grismer et al., 2007, 2008; Neang et al., 2010; Stuart & Emmett, 2006, Stuart et al., 2006, 2010) (see below for *S.* cf. *rupicola*).

**Figure 1:**
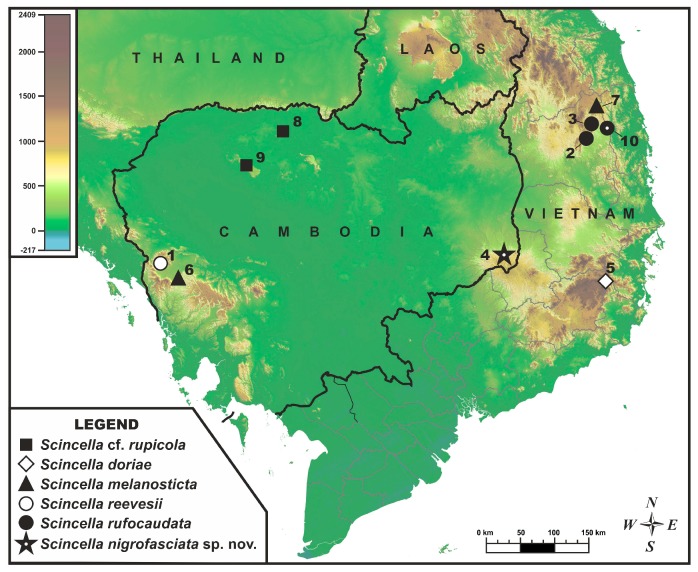
Map showing type locality of *Scincella nigrofasciata* sp. nov. at Keo Seima Wildlife Sanctuary, eastern plain, Cambodia, and locations of populations included in molecular analyses.

Following recent changes in and transfer of the protected area management from the Ministry of Agriculture, Forestry, and Fisheries to the Ministry of Environment of Cambodia, the Keo Seima Biodiversity Conservation Area was reorganized and renamed as the Keo Seima Wildlife Sanctuary, covering an area of 292 690 hectares and spanning the Mondulkiri and Kratie provinces of south-eastern Cambodia. The sanctuary is located in the Keo Seima, O’Raing, and Senmorom districts in Mondulkiri Province and Snoul District of Kratie Province in Cambodia (Figure 1). Despite its high biodiversity, low level of disturbance, and high percentage of forest cover (Nuttall et al., 2015), little is known about the sanctuary’s herpetofauna. Recent herpetological field surveys in Cambodia have focused on the Cardamom Mountains (Grismer et al., 2007, 2008; Neang et al., 2010, 2015; Stuart & Emmett, 2006), with only two undertaken in Mondulkiri Province (Neang & Poyarkov, 2016; Stuart et al., 2006). Biogeographically, the hilly areas of the eastern plain of Cambodia are linked to the Annamite Range (or Truong Son Mountains) of Vietnam (Poyarkov et al., 2017; Stuart et al., 2006), where many new herpetofaunal species have been described in recent years (Hartmann et al., 2013; Nazarov et al., 2012; Nguyen et al., 2013; Poyarkov et al., 2014, 2015a, 2015b; Rowley et al., 2016).

For locality information also see Table 2: 1: Phnom Samkos W.S., Pursat Province, southwest Cambodia; 2: Kon Ka Kinh N.P., southern sector, Gia Lai Province, Vietnam; 3: Kon Ka Kinh N.P., eastern sector, Gia Lai Province, Vietnam; 4: Keo Seima W.S., Mondulkiri Province, Cambodia (type locality of *Scincella nigrofasciata* **sp. nov.**); 5: Bidoup - Nui Ba N.P., Lam Dong Province, Vietnam; 6: Veal Veng District, Pursat province, Cardamom Mountains, Cambodia; 7: Kon Chu Rang N.R., Gia Lai Province, Vietnam; 8: Kuleaen District, Preah Vihear Province, Cambodia; 9: Phnom Kulen District, Krong Siem Reap, Cambodia; 10: Buon Luoi, Gia Lai Province, Vietnam (type locality of *Scincella rufocaudata*; not sampled). White dot indicates type locality.

During a field survey at Prey Lang in northern central Cambodia between June and July 2014, 10 specimens were collected and tentatively assigned to *Scincella* cf. *rupicola* based on their external morphology (Hayes et al., 2015; see Discussion). During a second herpetofaunal survey between 22 and 28 September 2016 in Keo Seima Wildlife Sanctuary in south-east Cambodia, we recorded nine species of amphibians and 17 species of reptiles. Among these, eight specimens were assigned to the genus *Scincella* based on their body habitus and external morphology. However, further morphological and molecular analyses indicated that this population represents a yet to be described species of *Scincella*, which we describe herein.

## MATERIALS AND METHODS

### Sampling

The herpetofauna field survey was undertaken during the day and night between 22 and 28 September 2015 in semi-evergreen forest in Keo Seima Wildlife Sanctuary. Specimens were captured by hand and kept in plastic bags until the next morning. Specimens were photographed prior to euthanasia and subsequent preservation in 10% formalin. Liver tissue samples were taken for molecular analyses prior to preservation in formalin and subsequently stored in 95% ethanol. Upon arrival to the collection, the specimens were washed in water for 12 h, then transferred to 70% ethanol for storage. Specimens were deposited in the Zoological Museum at the Centre for Biodiversity Conservation of the Royal University of Phnom Penh (CBC RUPP). Additionally, we examined the type series, including the holotype specimen of *Sphenomorphus rufocaudatus* Darevsky & Nguyen, 1983 (ZISP 19797, St. Petersburg, Russia).

### Morphological analyses

Characters were observed under a Nikon SMZ 645 dissecting microscope and measured with a digital caliper to the nearest 0.1 mm and ratio to 0.01. The following morphometric characters were measured: eye diameter (ED) – maximum horizontal diameter of eye; forearm length (FoL) – length between forelimb elbow and tip of fourth finger with limb held at right angle to body; forelimb length (FlL) – length between axilla and tip of fourth finger with limb held at right angle to body; head depth (HD) – maximum height posterior to extremity of eye; head length (HL) – length from tip of snout to posterior margin of parietals; hind limb length (HlL) – length from groin and tip of fourth toe with limb held at right angle to body; head width (HW) – maximum width of head; snout-forelimb length (SFlL) – length from snout to anterior margin of axilla; snout length (SnL) – length from anterior corner of eye to tip of snout; snout-tympanum length (STL) – length from snout to anterior margin of tympanum; snout to vent length (SVL) – length from tip of snout to vent; tail length (TaL) – length from vent to tip of tail; tympanum diameter (TD) – maximum diameter of ear; trunk length (TrunkL) – length from posterior margin of axilla to anterior groin, with limbs held at right angles to body. Scale counts included: supralabials (SL) – number of upper labial scales; infralabials (IL) – number of lower labial scales; temporals including primary temporals – number of scales above posterior supralabial, posterior postsuboculars, and below parietal and secondary temporal; supraciliaries – counted following Ouboter (1986) and Lim (1998); enlarged nuchals (EnLN) – number of enlarged nuchal scales contacting to parietals posteriorly; midbody scale rows (MBSR) – scales around midpoint of trunk; paravertebral scale rows (PVSR) – number of scales from posterior edge of parietals to point opposite vent; dorsal scale rows between dorsolateral stripes (DBR) – number of dorsal scale rows at midbody between dark dorsolateral stripes, following Inger et al. (1990); ventral scales (VS) – number of scales between gulars and preanal scales; subdigital lamellae under fourth finger (SDLF4); subdigital lamellae under fourth toe (SDLT4).

Morphological data used for comparisons were taken from previously published literature, namely, Taylor (1963); Darevsky & Nguyen (1983); Ouboter (1986); Darevsky & Orlov (1997); Gonzalez et al. (2005); Stuart et al. (2006); Stuart & Emmett (2006); Nguyen et al. (2010a, 2010b); Luu et al. (2013), and Pham et al. (2015) (Table 1), and from examination of museum specimens (Appendix I). Museum abbreviations include: CBC, Centre for Biodiversity Conservation, Royal University of Phnom Penh, Cambodia; ZISP, Zoological Institute, Russian Academy of Sciences, St. Petersburg, Russia; ZMMU, Zoological Museum of Moscow University, Moscow, Russia.

**Table 1:** Morphometric and meristic characters of *Scincella nigrofasciata* sp. nov.

**Characters**	**CBC02546**	**CBC02545**	**CBC02840**	**CBC02841**	**CBC02842**	**Min-Max**
	Holotype (F)	Paratype (M)	Paratype (F)	SubA	SubA	
SVL	52.6	50.2	50.6	42.0	40.0	40.0–52.6
TaL	84.0	97.3	63.0	65.7	broken	63.0–97.3
HL	8.5	8.9	8.3	7.1	6.9	6.9–8.9
HW	6.1	6.3	6.2	5.1	5.1	5.1–6.3
HD	4.2	4.5	4.4	4	3.8	3.8–4.5
SnL	3.4	3.8	3.2	3.0	3.0	3.0–3.8
STL	8.8	9.4	8.4	7.6	7.6	7.6–9.4
SFlL	16.9	17.8	15.8	15.0	14.0	14.0–17.8
TD	1.5	1.6	1.5	1.3	1.3	1.3–1.6
FoL	7.4	8.2	7.4	6.5	6.3	6.3–8.2
FoL/SVL	0.14	0.16	0.15	0.15	0.16	0.14–0.6
FIL	10.8	10.8	10.2	9.1	9.0	9.0–10.8
HIL	16.6	16.8	16.3	13.6	13.3	13.3–16.8
TrunkL	29.7	25.8	28.4	22.3	20.1	20.1–29.7
TaL/SVL	1.60	1.94	1.25	1.27	N/A	1.25–1.94
FIL/SVL	0.21	0.22	0.20	0.20	0.20	0.20–0.22
HIL/SVL	0.32	0.33	0.32	0.30	0.30	0.30–0.33
TrunkL/SVL	0.56	0.51	0.56	0.50	0.50	0.50–0.56
FIL/TrunkL	0.4	0.4	0.4	0.4	0.4	0.4
HIL/TrunkL	0.6	0.7	0.6	0.6	0.7	0.6–0.7
TrunkL/(FIL+HIL)	1.1	0.9	1.1	1.0	0.9	0.9–1.1
Adpressed limbs	–2.3	1.8	–1.9	0.4	2.2	–2.3–2.2
SL	6	7	7	7	7	6–7
IL	6	6	6	6	6	6
Supraciliaries	8	7	7	8	7	7–8
Prefrontal in contact	+	+	+	+	+	+
Supraoculars	4	2L–3R	4	4	4	2–4
Lower eyelids	Transparent window	Transparent window	Transparent window	Transparent window	Transparent window	Transparent window
Primary temporal	2	2	2	2	2	2
Upper secondary temporal enlarged	Yes	Yes	Yes	Yes	Yes	Yes
EnLN	Weakly enlarged	1	Weakly enlarged	Weakly enlarged	Weakly enlarged	0–1
Lobules on external ear opening	Absent	Absent	Absent	Absent	Absent	Absent
Smooth dorsal scales	Yes	Yes	Yes	Yes	Yes	Yes
MBSR	32	32	32	32	33	32–33
PRVSR	74	69	71	74	70	69–74
Ventral scales	69	65	68	69	65	65–69
Precloacals	2	2	2	2	2	2
Inner overlapping outers	Yes	Yes	Yes	Yes	Yes	Yes
DBR	8	8	8	8	8	8
SDLF4	11	11	10L–11R	11	11	10–11
SDLT4	16	16	15	17	16	15–17
Dorsal color	Dark brown	Dark brown	Dark brown	Dark brown	Dark brown	Dark brown
Dark vertebral stripe	Yes	Yes	Yes	Yes	Yes	Yes
Dorsal stripes	5–7	5–7	5–7	5–7	5–7	5–7
Upper flank (dorsolateral) bands	Distinct regular black	Distinct regular black	Distinct regular black	Distinct regular black	Distinct regular black	Distinct regular black
Pad and lamellae color	Dark grey	Dark grey	Dark grey	Dark grey	Dark grey	Dark grey
% of bifurcating hemipenis length	N/A	63%	N/A	N/A	N/A	63%

Abbreviation of character states: in contact (+); male (M), female (F), subadult (SubA); positive values in “Adpressed limbs” correspond to length of overlap between adpressed limbs (in mm); negative values correspond to length of gap separating finger tips of fore- and hindlimbs when adpressed (in mm), L (left); R (right).

### DNA isolation, PCR, and sequencing

For molecular analysis, we examined 22 specimens of *Scincella* from Cambodia and adjacent areas of Vietnam, with the sequence from *Sphenomorphus maculatus* used as an outgroup (Table 2). The geographic locations of the examined populations are shown in Figure 1.

**Table 2:** Specimens and sequences of *Scincella* representatives used in molecular analyses of the mtDNA *COI* gene fragments.

Museum Specimen ID	GenBank accession No.	Species	Locality
CBC01357	MH119607	*Scincella reevesii*	(1) Phnom Samkos W.S., Pursat Province, southwest Cambodia
CBC01358	MH119608	*Scincella reevesii*	(1) Phnom Samkos W.S., Pursat Province, southwest Cambodia
CBC01380	MH119609	*Scincella reevesii*	(1) Phnom Samkos W.S., Pursat Province, southwest Cambodia
CBC01379	MH119610	*Scincella reevesii*	(1) Phnom Samkos W.S., Pursat Province, southwest Cambodia
ZMMU NAP-06163	MH119611	*Scincella rufocaudata*	(2) Kon Ka Kinh N.P., southern sector, Gia Lai Province, Vietnam
ZMMU NAP-06164	MH119612	*Scincella rufocaudata*	(3) Kon Ka Kinh N.P., eastern sector, Gia Lai Province, Vietnam
CBC02545	MH119613	*Scincella nigrofasciata* **sp. nov.**	(4) Keo Seima W.S., Mondulkiri Province, Cambodia
CBC02546	MH119614	*Scincella nigrofasciata* **sp. nov.**	(4) Keo Seima W.S., Mondulkiri Province, Cambodia
ZMMU R-13268-00412	MH119616	*Scincella doriae*	(5) Bidoup - Nui Ba N.P., Lam Dong Province, Vietnam
ZMMU R-13268-00505	MH119615	*Scincella doriae*	(5) Bidoup - Nui Ba N.P., Lam Dong Province, Vietnam
ZMMU R-13268-01062	MH119617	*Scincella doriae*	(5) Bidoup - Nui Ba N.P., Lam Dong Province, Vietnam
CBC01431	MH119618	*Scincella melanosticta*	(6) Phnom Samkos W.S., Pursat Province, southwest Cambodia
CBC01808	MH119619	*Scincella melanosticta*	(6) Phnom Samkos W.S., Pursat Province, southwest Cambodia
CBC01430	MH119620	*Scincella melanosticta*	(6) Phnom Samkos W.S., Pursat Province, southwest Cambodia
ZMMU NAP-05519	MH119621	*Scincella melanosticta*	(7) Kon Chu Rang N.R., Gia Lai Province, Vietnam
ZMMU NAP-06376	MH119622	*Scincella melanosticta*	(7) Kon Chu Rang N.R., Gia Lai Province, Vietnam
S.r.-1 (no voucher)	MH119623	*Scincella* cf. *rupicola*	(8) Kuleaen District, Preah Vihear Province, Cambodia
S.r.-2 (no voucher)	MH119624	*Scincella* cf. *rupicola*	(8) Kulaeen District, Preah Vihear Province, Cambodia
S.r.-3 (no voucher)	MH119625	*Scincella* cf. *rupicola*	(8) Kulaeen District, Preah Vihear Province, Cambodia
S.r.-4 (no voucher)	MH119626	*Scincella* cf. *rupicola*	(8) Kuleaen District, Preah Vihear Province, Cambodia
S.r.-5 (no voucher)	MH119627	*Scincella* cf. *rupicola*	(9) Phnom Kulen District, Krong Siem Reap, Cambodia
S.r.-6 (no voucher)	MH119628	*Scincella* cf. *rupicola*	(9) Phnom Kulen District, Krong Siem Reap, Cambodia
ZMMU R-13680-00094	MH119629	*Sphenomorphus maculatus*	Cat Tien N.P., Dong Nai Province, Vietnam

N.P.: National Park; N.R.: Nature Reserve; W.S.: Wildlife Sanctuary. For locality numbers see Figure 1.

For molecular phylogenetic analyses, total genomic DNA was extracted from ethanol-preserved femoral muscle and liver tissues using standard phenol-chloroform-proteinase K (final concentration 1 mg/mL) extraction, with subsequent isopropanol precipitation (protocols per Hillis et al., 1996 and Sambrook et al., 1989). Isolated total genomic DNA was visualized by 1.5% agarose gel electrophoresis in the presence of ethidium bromide. The concentration of total DNA was measured in 1 μL using a NanoDrop 2000 (Thermo Scientific, USA), and consequently adjusted to 100 ng DNA/μL.

We amplified a 653-bp fragment of cytochrome oxidase I (*COI*), a mitochondrial marker widely used as a DNA-barcoding marker for vertebrates, including reptiles and amphibians (Murphy et al., 2013; Nagy et al., 2012; Smith et al., 2008), and for species identification in various groups of lizards (Amarasinghe et al., 2017; Hartmann et al., 2013; Nazarov et al., 2012, 2014; Orlova et al., 2017 Solovyeva et al., 2011, 2014). We used two primer pairs for PCR and sequencing, depending on their performance in PCR, for different samples. The first primer pair was VF1-d (5${}^{\prime }$-TTCTCAACCAACCACAARGAYATYGG-3${}^{\prime }$, forward primer) and VR1-d (5${}^{\prime }$-TAGACTTCTGGGTGGCCRAARAAYCA-3${}^{\prime }$, reverse primer) (Ivanova et al., 2006); the second primer pair was RepCOI-F (5${}^{\prime }$-TNTTMTCAACNAACCACAAAGA-3${}^{\prime }$, forward primer) and RepCOI-R (5${}^{\prime }$-ACTTCTGGRTGKCCAAARAATCA-3${}^{\prime }$, reverse primer) (Nagy et al., 2012). PCR arrays were performed in 25-μL reactions using 50 ng of genomic DNA, 10 pmol of each primer, 15 nmol of each dNTP, 50 nmol additional MgCl_2_, Taq PCR buffer (10 mmol/L Tris-HCl, pH 8.3, 50 mmol/L KCl, 1.1 mmol/L MgCl_2_, and 0.01% gelatin), and 1 U of Taq DNA polymerase. The PCR conditions for the *COI* gene fragment followed Nazarov et al. (2012) and included an initial denaturation step at 95 ${}^{\circ }$C for 3 min; 5 cycles at 95 ${}^{\circ }$C for 30 s, annealing at 45 ${}^{\circ }$C for 1 min, extension at 72 ${}^{\circ }$C for 2 min, followed with 35 cycles at 95 ${}^{\circ }$C for 30 s, annealing at 51 ${}^{\circ }$C for 1 min, extension at 72 ${}^{\circ }$C for 2 min, and final extension at 72 ${}^{\circ }$C for 5 min.

The PCR products were loaded onto 1.5% agarose gels in the presence of ethidium bromide and visualized by agarose electrophoresis. If distinct bands were produced, products were purified using 2 μL from a 1:4 dilution of ExoSap-It (Amersham, UK) per 5 μL of PCR product prior to cycle sequencing. The 10-μL sequencing reaction included 2 μL of template, 2.5 μL of sequencing buffer, 0.8 μL of 10 pmol primer, 0.4 μL of BigDye Terminator v3.1 Sequencing Standard (Applied Biosystems, USA), and 4.2 μL of water. The cycle sequencing reaction consisted of 35 cycles of 10 s at 96 ${}^{\circ }$C, 10 s at 50 ${}^{\circ }$C, and 4 min at 60 ${}^{\circ }$C. Cycle sequencing products were purified by ethanol precipitation. Sequence data collection and visualization were performed on an ABI 3730xl automated sequencer (Applied Biosystems, USA). The obtained fragments were sequenced in both directions for each sample, and a consensus sequence was generated using SeqMan v5.06 (Burland, 1999). The obtained sequences were deposited in GenBank under accession numbers MH119607–MH119629 (Table 2).

### Phylogenetic analyses

The *COI* dataset subjected to phylogenetic analyses included 22 *Scincella* representatives from Cambodia and Vietnam and *Sphenomorphus maculatus* used as an outgroup to *Scincella* based on Pyron et al. (2013) (Table 2).

Nucleotide sequences were initially aligned using ClustalX 1.81 (Thompson et al., 1997) with default parameters, and then optimized manually in BioEdit 7.0.5.2 (Hall, 1999) and MEGA 7.0 (Kumar et al., 2016). The final alignment included 668 sites. Mean uncorrected genetic distances (*P*-distances) between sequences were determined with MEGA 7.0. MODELTEST v.3.06 (Posada & Crandall, 1998) was used to estimate the optimal model of DNA evolution. The best-fitting models selected for the *COI* dataset were SYM+I for the first, F81+I for the second, and HKY+G for the third codon positions, as suggested by the Akaike Information Criterion (AIC).

Phylogenetic trees were inferred using Bayesian inference (BI) and maximum likelihood (ML). BI was conducted in MrBayes 3.1.2 (Huelsenbeck & Ronquist, 2001; Ronquist & Huelsenbeck, 2003); Metropolis-coupled Markov chain Monte Carlo (MCMCMC) analyses were run with one cold chain and three heated chains for four million generations and sampled every 1 000 generations. Five independent MCMCMC runs were performed and 1 000 trees were discarded as burn-in. We checked the convergence of the runs and that the effective sample sizes (ESS) were all above 200 by exploring the likelihood plots using TRACER v1.5 (Rambaut & Drummond, 2007). Confidence in tree topology was assessed by posterior probabilities (BPP) (Huelsenbeck & Ronquist, 2001). The ML analyses were conducted using Treefinder (Jobb et al., 2004). Confidence in tree topology was tested by non-parametric bootstrap analysis (MLBS) with 1 000 replicates (Felsenstein, 1985). We *a priori* regarded tree nodes with bootstrap (MLBS) values of 70% or greater and posterior probabilities (BPP) values over 0.95 as sufficiently resolved, those MLBS between 70% and 50% (BPP between 0.95 and 0.90) as tendencies, and those MLBS below 50% (BPP below 0.90) as unresolved (Felsenstein, 2004; Huelsenbeck & Hillis, 1993).

## RESULTS

### Molecular differentiation of *Scincella* species in Cambodia

#### Sequence data

Final alignment of the examined mtDNA *COI* gene fragments consisted of 668 sites, with 445 conserved sites and 223 variable sites, of which 220 were parsimony-informative. The transition-transversion bias (R) was 4.59. Nucleotide frequencies were 24.16% (A), 29.21% (T), 27.82% (C), and 18.81% (G) (data given for ingroup only).

### Genealogical relationships and species identification inferred from *COI* dataset

**Figure 2:**
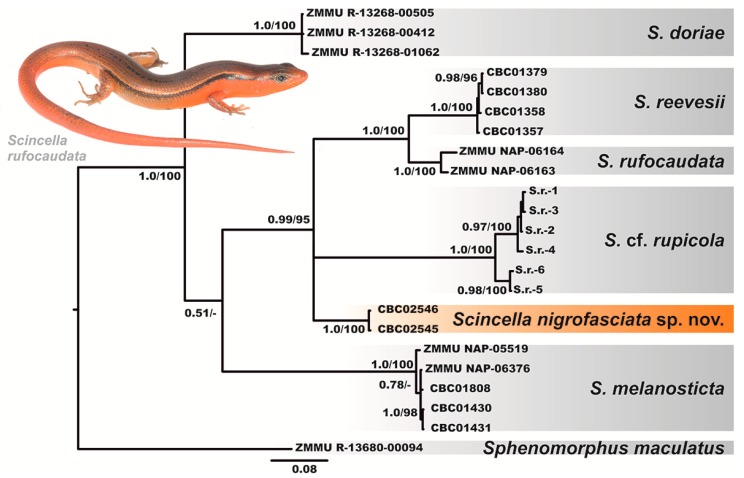
Bayesian inference dendrogram of *Scincella* derived from analysis of a 668-bp fragment of mtDNA *COI* gene.

The BI and ML analyses showed essentially similar topologies (Figure 2), differing only slightly from each other in associations at several poorly supported basal nodes. All six examined species of *Scincella* formed six corresponding clades with high levels of node support (BPP=1.0; MLBS=100%).

Voucher specimen IDs and GenBank accession numbers are given in Table 2. *Sphenomorphus maculatus* was used as an outgroup. Numbers near nodes represent posterior probability (BPP) or bootstrap support values (MLBS, 1 000 replicates) for BI/ML inferences, respectively.

The partial *COI* gene fragment can be applied as a DNA-barcoding marker, but should not be used as a single tool for reconstructing phylogenetic relationships (Murphy et al., 2013). However, the examined fragment clearly showed that *S. reevesii* from the Cardamom Mountains in Cambodia, *S. rupicola* from central Cambodia, *S. rufocaudata* from central Vietnam, and the newly discovered population of *Scincella* from Mondulkiri Province formed a well-supported clade (BPP=0.99; MLBS=95%), though phylogenetic relationships within this clade were essentially unresolved. *Scincella reevesii* from Cardamom Mountains and *S. rufocaudata* from central Vietnam were phylogenetically close to each other and represented sister species in our analyses (Figure 2). There was slight differentiation within *S. rupicola*, which clustered in two reciprocally monophyletic groups.

### Genetic distances

The uncorrected genetic *P*-distances in the examined *COI* gene fragments among and within the studied *Scincella* species are shown in Table 3.

**Table 3:** Genetic divergence between and within the examined *Scincella* species

Species		1	2	3	4	5	6	7
**1**	*Scincella nigrofasciata* **sp. nov.**	**0.16**	1.43	1.33	1.49	1.50	1.48	1.66
**2**	*Scincella reevesii*	15.96	**0.37**	1.02	1.40	1.52	1.64	1.70
**3**	*Scincella rufocaudata*	13.29	8.84	**2.99**	1.35	1.52	1.66	1.74
**4**	*Scincella doriae*	17.48	21.12	19.21	**0.63**	1.57	1.57	1.71
**5**	*Scincella melanosticta*	19.92	18.43	19.26	18.82	**0.41**	1.62	1.70
**6**	*Scincella* cf. *rupicola*	16.72	20.70	19.05	20.00	21.58	**2.54**	1.65
**7**	*Sphenomorphus maculatus*	18.48	21.78	20.68	20.13	21.76	19.94	—

Uncorrected *P*-distances (percentages) between *COI* sequences of *Scincella* species included in phylogenetic analyses (below diagonal) and standard error estimates (above diagonal). Ingroup mean uncorrected interspecific *P*-distances are shown on the diagonal.

The observed interspecific distances in the *COI* gene between the examined *Scincella* species varied from *P*=8.84% (between *S. reevesii* and *S. rufocaudata*) to *P*=21.58% (between *S. rupicola* and *S. melanosticta*) (Table 3). The observed intraspecific distances in our analysis varied from *P*=0.16% to *P*=2.99%, with the latter value corresponding to genetic differentiation between mtDNA lineages of *S. rufocaudata* (Table 3).

### Systematics

The newly discovered population of *Scincella* from Mondulkiri Province represents an independent mtDNA lineage, with phylogenetic relationships to *S. reevesii*, *S. rufocaudata*, and *S. rupicola* (Figure 2). This population was clearly distinct in *COI* sequences from all examined congeners with *P*-distances in interspecific comparisons varying from 13.29% (with *S. rufocaudata*) to 19.92% (with *S. melanosticta*) (Table 3), indicating deep divergence in the examined mtDNA marker.

Morphologically the Mondulkiri population of *Scincella* also showed affinities with *S. reevesii* and *S. rufocaudata*; however, it can be easily diagnosed from these species and other congeners inhabiting the Indochina region by several morphological diagnostic characters (see Comparisons below). Herein, we describe this population as a new species.

*Scincella nigrofasciata* **sp. nov.**

Figure 1, Figure 2, Figure 3, Figure 4, Figure 5, Figure 6, Figure 7 and Figure 8; Table 1, Table 2, Table 3, Table 4, Table 5 and Table 6. 

**Figure 3:**
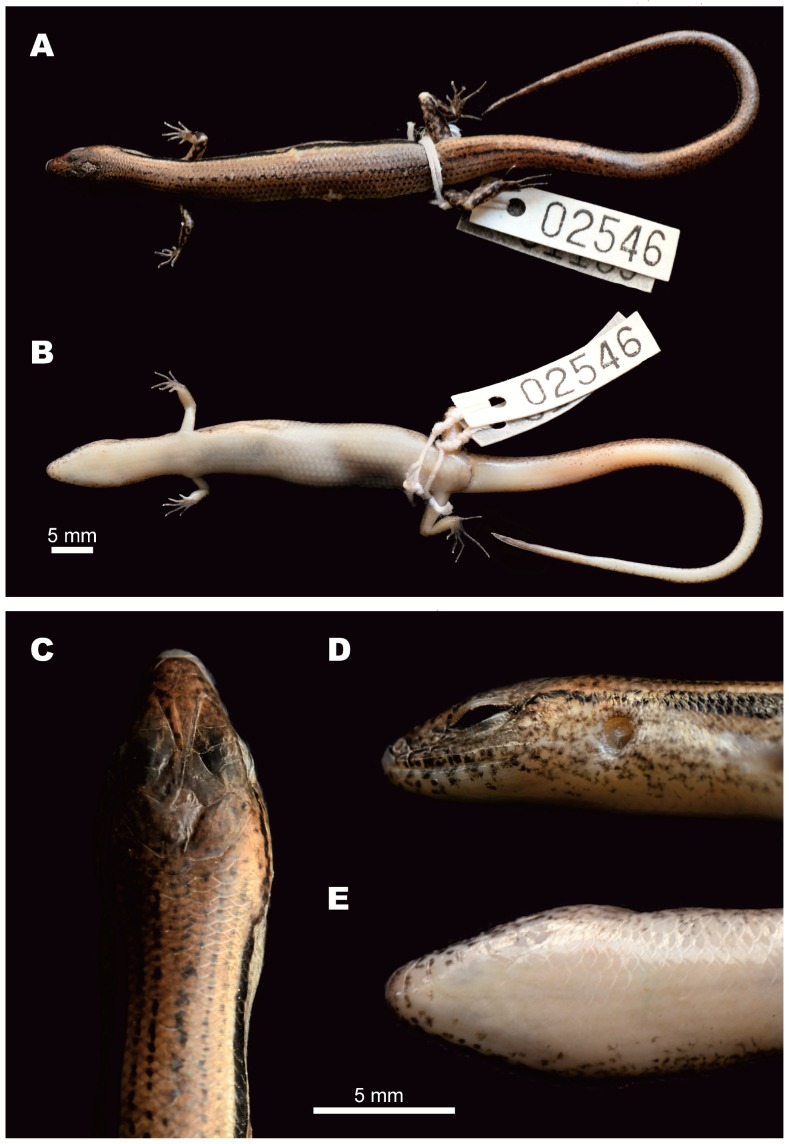
Female holotype of *Scincella nigrofasciata* sp. nov. (CBC02546) in preservative.

A: Dorsal view; B: Ventral view; C: Dorsal view of head; D: Lateral view of head; E: Ventral view of head. Scale bar: 5 mm. Photos by Thy Neang.

**Figure 4:**
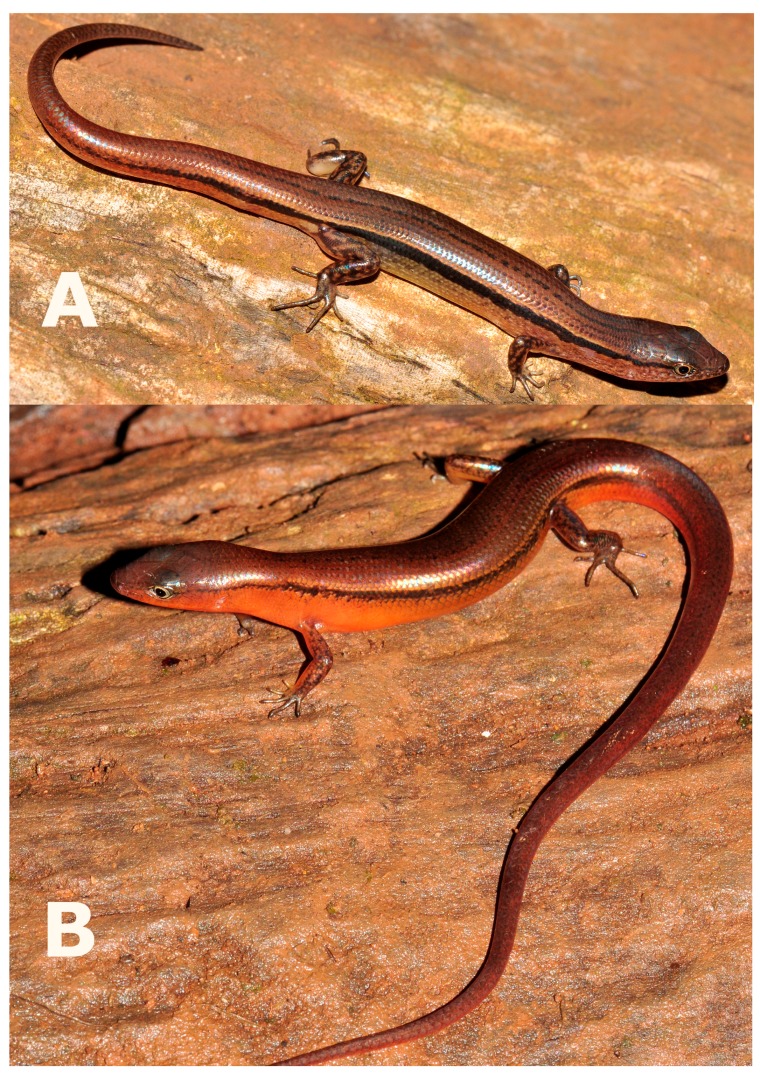
Coloration in life of *Scincella nigrofasciata* sp. nov. (Photos by Thy Neang).

A: Female paratype (CBC02840); B: Male paratype (CBC02545).

**Figure 5:**
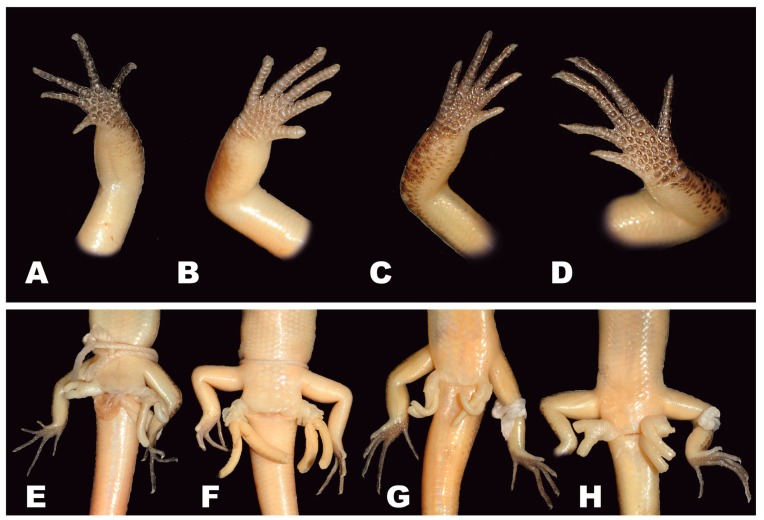
Morphology and coloration of fingers (A–D) and structure of hemipenes (E–H) of Cambodian *Scincella* species (Photos by Thy Neang).

A, E: Male paratype (CBC02545, SVL 50.2 mm) of *Scincella nigrofasciata* **sp. nov.**; B, F: Male (CBC02409, SVL 53.1 mm) of *S. rupicola*; C, G: Male (CBC1342, SVL 41.8 mm) of *S. reevesii*; D, H: Male (CBC01434, SVL 49.4 mm) of *S. melanosticta*.

**Figure 6:**
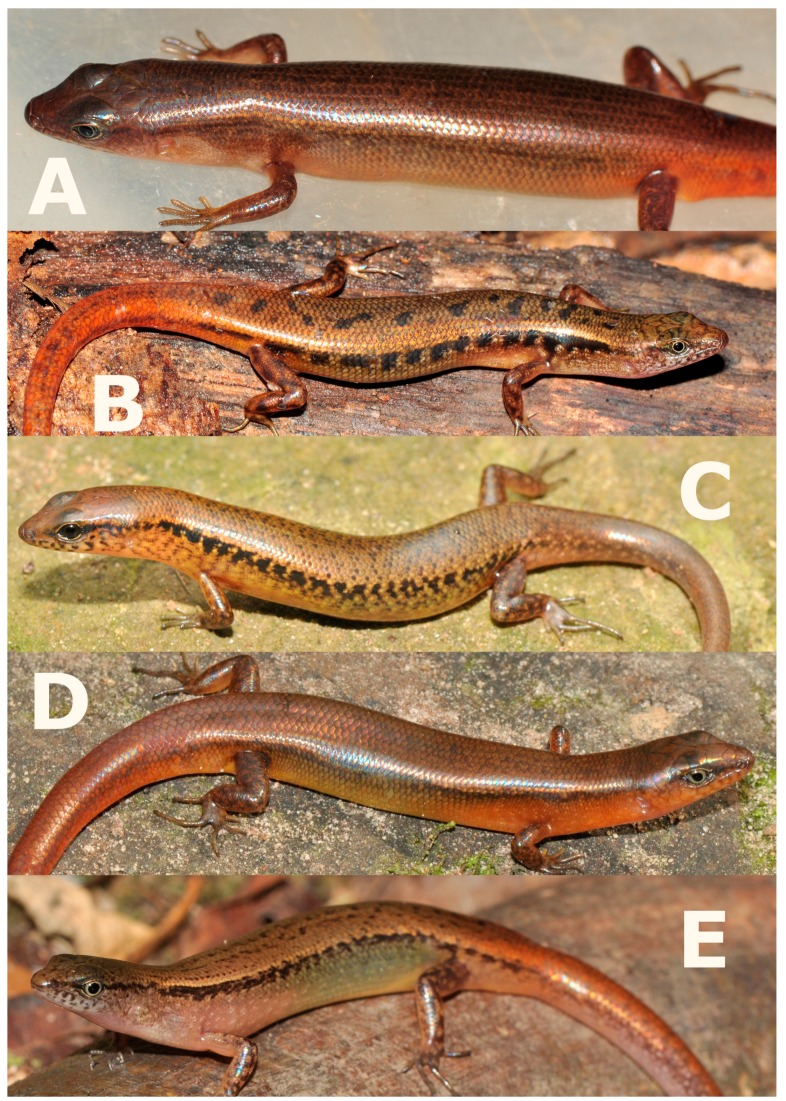
Differences in dorsal and dorsolateral views in life between *Scincella* species from Cambodia (Photos by Thy Neang).

A: Male of *S. rupicola* (CBC02409); B: Female of *S. rupicola* (CBC02339), both A and B from central part (Prey Lang) of Cambodia; C: Male of *S. melanosticta* (CBC01009); D: Male of *S. reevesii* (CBC02305); E: Female of *S. reevesii* (CBC01382); C, D, and E from Phnom Samkos Wildlife Sanctuary, Cardamom Mountains, southwest Cambodia.

**Figure 7:**
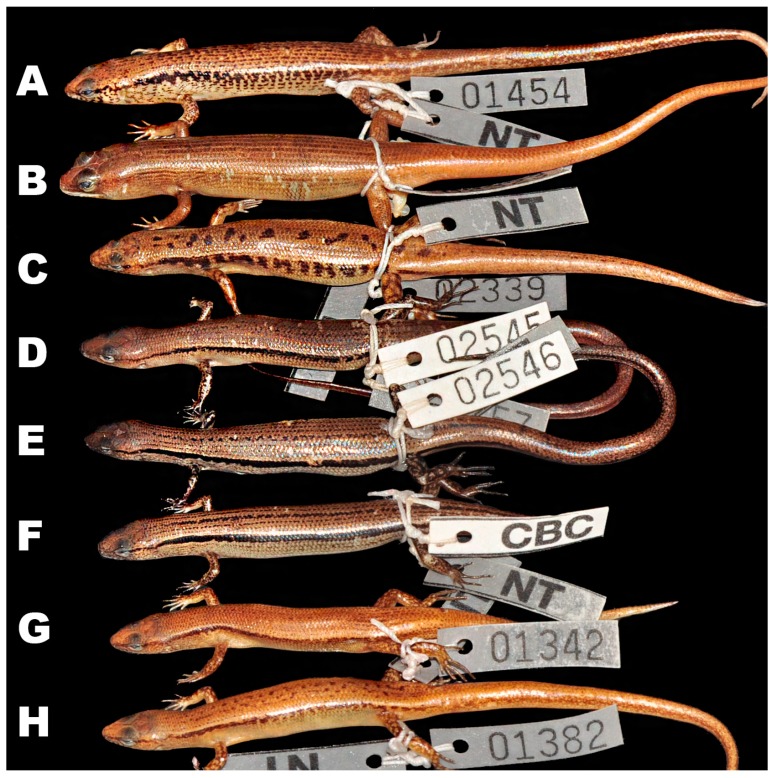
Dorsolateral views of representatives of Cambodian *Scincella* species (in preservative) (Photos by Thy Neang).

A: Male (CBC01454) of *S. melanosticta*; B: Male (CBC02409) and C: Female (CBC02339) of *S. rupicola*; D: Male paratype (CBC02545), E: Female paratype (CBC02840), and F: Female holotype (CBC02546) of *Scincella nigrofasciata* **sp. nov.**; G: Male (CBC1342) and H: Female (CBC01382) of *S. reevesii*.

**Figure 8:**
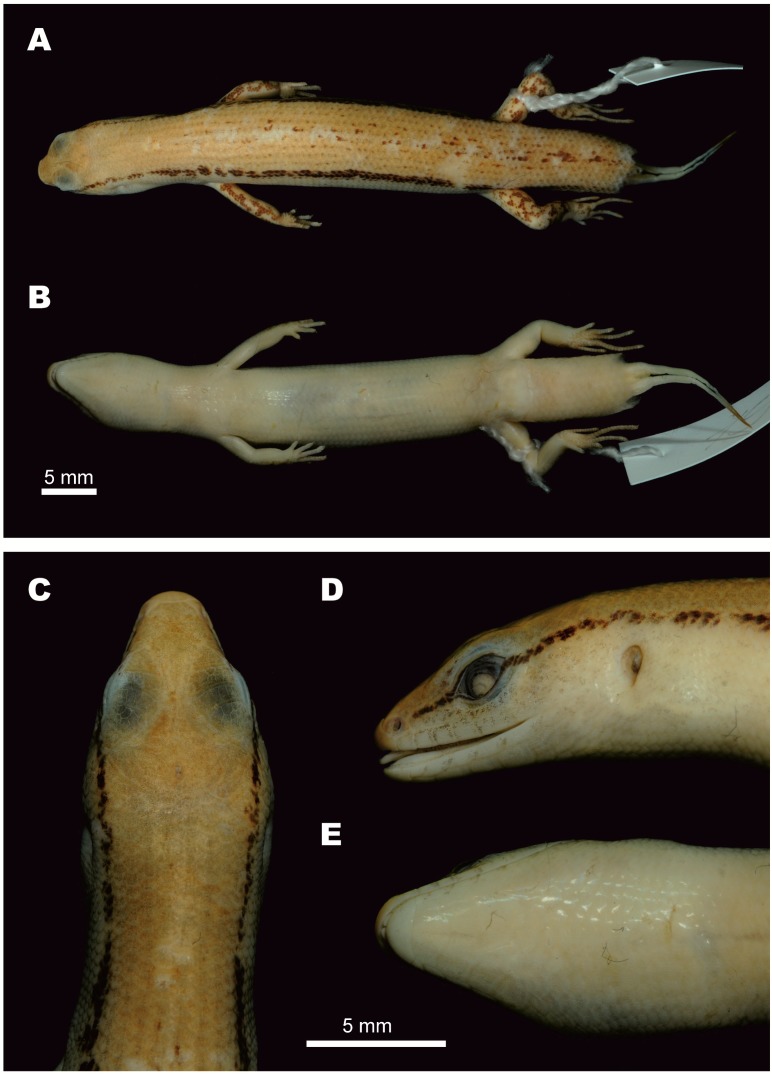
Male holotype of *Sphenomorphus rufocaudatus* (Darevsky & Nguyen, 1983) (ZISP 19797; now *Scincella rufocaudata*) (Photos by Nikolay A. Poyarkov).

A: Dorsal view; B: Ventral view; C: Dorsal view of head; D: Lateral view of head; E: Ventral view of head. Scale bar: 5 mm.

**Table 4:** Comparison of diagnostic morphometric and meristic characters of the new species and other Scincella species of Southeast Asia

Selected characters	*Scincella nigrofasciata* sp. nov.	*S. apraefrontalis*	*S. darevskii*	*S. devorator*	*S. doriae*	*S. melanosticta*	*S. monticola*	*S. ochracea*	*S. punctatolineata*	*S. reevesii*	*S. rufocaudata*	*S. rara*	*S. rupicola*	*S. victoriana*
SVL	40.0–52.6	36.1	88.6	44.4–60.7	58.6	47.4–57.4	45.4	42.9–51	37.6–40.2	49.1–58.0	50.7	45.0	51.1–53.6	41.4–76.7
FIL	9.0–10.8	5.1	18.5	17–17.2	N/A	11.4–13.3	7.0	9.2–10.4	6.5–7.1	10.5–12.3	11.0	N/A	11.4–13.8	N/A
HIL	13.3–16.8	8.1	28.6	21.6–22.1	N/A	17.2–20.8	8.9	12.7–16.6	8.7-11.4	15.3–17.8	18.7	N/A	16.8–21.3	14.6–23.9
TrunkL	20.1–29.7	21.4	49.5	27.3–28	N/A	22.9–25.9	18.3	26.1–30.0	23.0	20.3–24.1	27.9	20.0	24.6–29.2	21.1–44.2
FIL/SVL	0.20–0.22	0.14	0.21	0.32–0.33	N/A	0.23–0.27	0.21	0.17–0.27	0.17–0.19	0.24–0.30	0.22	N/A	0.23–0.26	N/A
HIL/SVL	0.30–0.33	0.22	0.32	0.40–0.42	N/A	0.35–0.39	0.28–0.29	0.30–0.38	0.25	0.34–0.43	0.37	N/A	0.36–0.40	0.29–0.35
Limb adpressed	Over/Sep	Sep	Sep	Sep	N/A	Over	Sep	Sep	Sep	Over	Over	Sep	Over	N/A
SL	6–7	6	7	7	N/A	7	7	7–8	7	7	7	7	7	7
IL	6	5	7	6	N/A	6	6	6	N/A	6	6–7	6	6	6
Supraciliaries	7–8	6	7	7–8	6–7	8–9	6	7–8	6–8	7–9	8	N/A	7–9	5–7
Prefrontal in contact	+	Absent*	0	0	0	0/+	Absent*	+	0	+	0	+	+	N/A
Supraoculars	2–4	4	5	4	4	4	4	4	4	4	4	4	4	N/A
Primary temporal	2	1	1	1	N/A	1	1	2	1	2	2	1	2	N/A
EnLN	0–1 pair	2–3 pairs	3 pairs	3	3–5 pairs	0	3–4 pairs	0–3 pairs	0–2 pairs	2	0	3 pairs	0–1 pair	3 pairs
Lobules on external ear opening	0	0	3 lobules	0	0	0	0	2–4 lobules	N/A	0–3 lobules	0	N/A	0	N/A
Dorsal scales	Smooth	Smooth	Smooth	Smooth	Smooth	Smooth	Smooth	Smooth	Smooth	Smooth	Smooth	Smooth	Smooth	Keeled
MBSR	32–33	18	28	28	30–32	34–37	22–26	30–32	22–28	29–35	30–34	24	33–36	26
PRVSR	69–74	52	62	63–66	66–76	63–73	52–59	61–67	58–69	60–71	67–69	53	68–73	50–57
VS	65–69	50	65	61–66	70–79	63–72	52–58	66–71	58–69	57–73	60-67	N/A	63–69	53–56
DBR	8	4	6	6	N/A	10	4	8	N/A	8	10	N/A	8	N/A
SDLT4	15–17	8–9	17	17–19	15–18	16–22	10–13	15–18	13–15	15–19	15	11	18–21	15–16
Dorsal color	Dark brown	Bronze-brown	Bronze	Bronze-brown	Caramel brown	Dark brown	Bronze-brown	Silver gray	Light brown	Brown with	Ochre	Golden brown	Dark brown	Dark brown
Dorsal pattern	5–7 regular discontinuous stripes	Faint dark spots	N/A	2 silver grey stripes, wide dark stripes	Brown spots	Irregular dark spots	Dark spots	Dark stripe	6 dark stripes	Irregular dark spots	1-3 dark brown spots	Irregular dark spots	Dark blotches	Dark spots
Light laterodorsal stripe	+	N/A	+	Large	N/A	+	0	Silver gray	N/A	+	Faint	Light spots	+	Whitish line
Upper flank dark stripe pattern	Wide black stripe	Indistinct dark spots	Dark brown	Dark-light mottling	With numerous whitish spots	Light dorsolateral bar broken with large dark blotches	Dark stripe with light spots	Black with light spots	Preset on upper and lower flanks	Irregular dark spots	Broken black stripe	Dark stripe	Dark blotches	Dark brown stripe
% of bifurcated hemipenis	63%	N/A	N/A	N/A	N/A	56%	N/A	N/a	N/A	65–68%	N/A	N/A	69–77%	N/A

Character states were taken from: Taylor (1963); Darevsky and Nguyen (1983); Ouboter (1986); Darevsky and Orlov (1997); Gonzalez et al. (2005); Stuart et al. (2006); Stuart and Emmett (2006); Nguyen et al. (2010a, 2010b, 2010c); Luu et al. (2013); Pham et al. (2015); and from specimens examination (see Appendix I). Abbreviation of character states: present or in contact (+); absent or not in contact (0); absent or damaged (absent*); separated (Sep); overlapping (Over); data not available (N/A).

**Table 5:** Morphometric and meristic character comparisons between *Scincella* species from Cambodia and *Scincella rufocaudata* from Vietnam

Characters	*Scincella nigrofasciata* sp. nov.	*S. melanosticta*	*S. reevesii*	*S. rufocaudata*	*S. rupicola*
SVL	40.0–52.6	47.4–57.4	38.3–57.4	50.7	46.4–55.2
TaL	63.0–97.3	63.5–88.9	64.8–71.6	–	53.6–81.2
HW	5.1–6.3	6.3–7.5	5.7–6.9	6.3	6.2–7.4
FIL	9.0–10.8	11.4–13.3	10.5–12.3	11.0	11.4–13.8
HIL	13.3–16.8	17.2–20.8	15.3–17.8	18.7	16.8–21.3
TrunkL	20.1–29.7	22.9–25.9	20.3–24.1	27.9	24.6–29.2
TaL/SVL	1.25–1.94	1.18–1.81	1.57–1.73	–	1.06–1.53
FIL/SVL	0.20–0.22	0.23–0.27	0.24–0.30	0.22	0.23–0.26
HIL/SVL	0.30–0.33	0.35–0.39	0.34–0.43	0.37	0.36–0.40
FoL	6.3–8.2	8.2–10.6	6.7–8.2	7.6	8.7–9.4
FoL/SVL	0.14–0.16	0.17–0.20	0.17–0.19	0.15	0.17–0.20
Adpressed limbs	–2.3–2.2	4.5–8.2	3.9–6.5	1.8	3.5–7.2
Supraciliaries	7–8	8–9	8–9	8	7–9
SL	6–7	7	7	7	7
IL	6	6	6	7	6
Prefrontals in contact	+	+	(0/+)	0	+
Supraoculars	2–4	4	4	4	4
Primary temporals	2	1	2	2	2
EnLN	0–1 pair	0	0–1 pair	0	0–1 pair
MBSR	32–33	34–37	29–35	32	33–36
PRVSR	69–74	63–73	60–71	68	68–73
VS	65–69	63–72	57–73	63	63–69
DBR	8	10	8	10	8
SDLT4	15–17	21–22	15–21	15	18–21
Dorsal color	Dark brown with 5–7 dark stripes	Dark brown with dark dense spots	Dark brown with irregular dark vertebral spots	Ochre with 1–3 dark brown spots	Dark brown with dark broken vertebral blotches and dark nuchal paired spots
Light dorsolateral stripe	Distinct	Faint	Distinct	Faint	Distinct in female
Upper flank pattern	Black band	Light dorsolateral bar broken with large dark blotches	Irregular dark spots	Distinct regular, broken black band, ending at tail base	Dark blotches
Pad and lamellae color	Dark grey	Light grey	Light grey	Dark grey	Dark grey
% of bifurcated hemipenis length	63%	56%	65–68%	N/A	69–77%
Texture of hemipenis	Smooth	Groove ring	Groove ring	N/A	Groove ring
Hemipenis thickness	Thick	Thick	Slender	N/A	Thick
Finger and toes	Slender	Thick	Thick	N/A	Thick
Body habitus	Stout	Stout	Slender	N/A	Stout

Characters of *S. rufocaudata* were obtained from Darevsky & Nguyen (1983), Luu et al. (2013), and from examination of its holotype specimen. Abbreviation of character states: present or in contact (+); absent or not in contact (0); data not available (N/A).

**Table 6:** Morphometric and meristic characters of the holotype of *Sphenomorphus rufocaudatus* Darevsky & Nguyen, 1983 (ZISP 19797; now *Scincella rufocaudata*)

Characters	ZISP 19797 Holotype (M)	Characters	ZISP 19797 Holotype (M)
SVL	50.7	Supraciliaries	8
TaL	85* broken	Prefrontals in contact	0
HL	9.0	Supraoculars	4
HW	6.3	Lower eyelids	Transparent window
HD	5.0	Primary temporals	2
SnL	3.6	Upper secondary temporal enlarged	No
STL	9.4	EnLN	Weakly enlarged
SFlL	19.1	Lobules on external ear opening	0
TD	1.4	Smooth dorsal scales	yes
FoL	7.6	MBSR	32
FoL/SVL	0.15	PRVSR	68
FIL	11.0	VS	63
HIL	18.7	Precloacals	2
TrunkL	27.9	Inner overlapping outers	Yes
TaL/SVL	1.7	DBR	10
FIL/SVL	0.22	SDLF4	10
HIL/SVL	0.37	SDLT4	15
TrunkL/SVL	0.55	Dorsal color	Ochre
FIL/TrunkL	0.4	Dark vertebral stripe	Indistinct paravertebral
HIL/TrunkL	0.7	Dorsal stripes	1–3
TrunkL/(FIL+HIL)	0.9	Upper flank (dorsolateral) bands	Distinct regular, broken black bands, ending at tail base
Adpressed limbs	0.21	Pad and lamellae color	Dark grey
SL	7	% of bifurcating hemipenis length	N/A
IL	7				

Abbreviation: Present or in contact (+); absent or not in contact (0); data not available (N/A). *: As the holotype has a broken tail, TaL is given according to the original description (Darevsky & Nguyen, 1983). M: male. For abbreviations, see “Materials and methods”.

**Holotype:** CBC02546, adult female, collected by Thy Neang on 25 September 2016 at N12${}^{\circ }$19${}^{\prime }$12.3${}^{\prime \prime }$, E107${}^{\circ }$04${}^{\prime }$20.8${}^{\prime \prime }$, 508 m a.s.l in Keo Seima Wildlife Sanctuary, O’Raing District, Mondulkiri Province, Cambodia. 

**Paratypes:** CBC02545, adult male, CBC02840, adult female, and CBC02841–42, two subadults, collected by Thy Neang at the same date and locality as given for the holotype.

**Referred materials:** CBC02843–45, three juveniles, collected by Thy Neang at the same date and locality as given for the holotype.

**Diagnosis:** The new species was assigned to the genus *Scincella* Mittleman, 1950 as it shows morphometric and meristic characters matching the diagnosis for this genus. *Scincella nigrofasciata* **sp. nov.** can be diagnosed from other congeners by the following combination of morphological attributes: (1) slender and medium-sized, SVL 40.0–52.6 mm; (2) tail relatively long, TaL/SVL (1.25–1.94); (3) FIL/SVL 0.20–0.22; (4) HIL/SVL 0.30–0.33; (5) forelimbs and hind limbs either slightly overlapping (0.4–2.2 mm) or slightly separated (1.9–2.3 mm) when adpressed to body toward each other; (6) infralabials 6; (7) supraciliaries 7–8; (8) prefrontals in broad contact; (9) primary temporals 2; (10) nuchal scales weakly enlarged; (11) external ear opening without lobules; (12) dorsal scales smooth: MBSR 32–33, PRVSR 69–74, VS 65–69, DBR 8; (13) SDLT4 15–17; (14) coloration pattern with dorsum dark brown/greyish-brown in life with 5–7 regular discontinuous dorsal dark stripes (formed by series of dark dots or elongated black spots), including paravertebral stripes, wide black dorsolateral stripes, 2–3 scale rows in width, starting from posterior corner of eye and continuing to lateral side of tail, extending 52%–86% of total tail length; and (15) hemipenis bifurcating about 63% of its total length to base. 

**Description of holotype** (Figure 3): A gravid adult female, SVL 52.6 mm; tail relatively long, TaL 84 mm, (TaL/SVL 1.6); head elongated, HL 8.5 mm (HL/SVL 0.16), longer than wide, HW 6.1 mm (HW/HL 0.72), slightly depressed, HD 4.2 mm (HD/HL 0.49). Neck rather slender, slightly distinct from head.

**Head:** Snout rounded in profile and dorsal view, SnL 3.4 mm, more than twice as long as TD (1.5 mm); STL 8.8 mm; SFlL 16.9 mm, comprising about one third of SVL; ear vertically oval, TD 1.5 mm; ED 2.5 mm; diameter of cornea 1.3 mm; rostral broad, (width 1.7 mm), almost three times greater than height (0.6 mm), visible from above, in contact with 1^st^ SL laterally, nasals and frontonasal posteriorly; supranasals absent; frontonasal broad, subtrapezoidal in shape, anterior side forming almost straight suture (0.6 mm) with rostral, posterior width 1.7 mm, as wide as rostral, little more than twice as wide as length (0.8 mm), in contact with nasals and 1^st^ loreal laterally, posterior margin slightly overlapping prefrontals; prefrontals in broad contact, laterally bordered by two loreals, frontal posteriorly; frontal elongated (length 2.8 mm), kite-shaped, posterior part much longer than anterior; greatest width anteriorly 1.4 mm, twice as narrow as length (width/length 0.5); frontal in contact with 1^st^ and 2^nd^ supraoculars laterally, frontoparietals posteriorly, anterior corner of rostral end slightly separating posterior portions of prefrontals medially, posterior corner of frontal slightly overlapping medial suture between frontoparietals; frontoparietals two, each diamond-shaped, together forming a butterfly-shape with median suture 1.2 mm, in contact with 2^nd^, 3^rd^, and 4^th^ supraoculars laterally, interparietal and parietals posteriorly; interparietal rather small, kite-shaped, with posterior portion little longer than anterior, in contact with parietals posteriorly, anterior corner of interparietal acute, slightly intruding into median suture between frontoparietals; parietals large, in contact with each other posteriorly (suture 0.6 mm behind posterior corner of interparietal), narrowly contacting 4^th^ supraocular and posterior supraciliary scale, in broad contact with upper secondary temporal laterally and four nuchal scales posteriorly. Naris rounded, laterally pierced in nasal scale; nasals in contact with 1^st^ SL ventrally, frontonasal dorsally, 1^st^ loreal posteriorly; loreals two, anterior loreal rhomboidal, in contact with 2^nd^ SL ventrally, frontonasal and prefrontal dorsally, posterior loreal subtrapezoidal, in contact with 2^nd^ and 3^rd^ SL ventrally, preocular and upper presubocular posteriorly, prefrontal and anterior supraciliary scale dorsally; preocular one, elongate, triangular, in contact with anterior supraciliary scale dorsally, anterior edge of orbit posteriorly, anterior presubocular ventrally; supraciliaries eight, anterior two largest; supraoculars four, first two contacting frontal, second to third contacting frontoparietal; presuboculars three, posterior-most slightly intruding into suture between 3^rd^ and 4^th^ SL, anterior-most triangular, slightly larger than posterior-most presubocular; suboculars two, both contacting 4^th^ SL, anterior slightly overlapping posterior one, slightly overlapped by lower presubocular, posterior broadly overlapping lower postsubocular, both bordered above by granular scales of lower eyelid; postoculars two; postsuboculars four (left side) and five (right side), lowest slightly intruding between 4^th^ and 5^th^ SL, uppermost largest; lower eyelid with distinct transparent disc (window) bordered above by small palpebral scales; supralabials six, 1^st^ smallest, 4^th^ located ventral to window of eye, 5^th^ largest; infralabials six, 1^st^ smallest, 5^th^ largest; primary temporals two, lower larger, sub-rhomboid, anteriorly in contact with 3^rd^ and 4^th^ postsuboculars, ventrally with 5^th^ and 6^th^ SL, posteriorly with lower secondary temporal, upper primary temporal subrhomboid, anteriorly in contact with 1^st^ and 2^nd^ postsubocular anteriorly, posteriorly with both secondary temporals; secondary temporals two, lower smaller, overlapping upper, in contact with 6^th^ SL ventrally, upper secondary temporal about twice as large as lower, in contact with posterior-most postsubocular anteriorly, with parietal dorsally and nuchal scale posteriorly; nuchal scales four, bordering posterior edge of parietals, slightly enlarged in comparison with adjacent posterior scales. Mental rounded, width (1.6 mm) more than twice as wide than long (0.7 mm), in contact with 1^st^ IL laterally, postmental posteriorly; postmental large, width (2.0 mm) greater than length (1.1 mm), in contact with 1^st^ and 2^nd^ IL laterally, 1^st^ chinshield posteriorly; chinshields in three pairs, 1^st^ pair in broad contact median with each other, contacting 3^rd^ IL laterally, 2^nd^ pair separated by subtriangular gular scale, contacting 4^th^ IL laterally, 3^rd^ pair separated medially by three gular scales, in contact with 5^th^ and 6^th^ IL laterally and three gular scales posteriorly.

**Body, limbs, and tail:** Body scales smooth, cycloid, imbricate; dorsal scales between dorsal stripes ½+8+½, same size as ventral scales, slightly larger than those on body sides and gular scales; scales on anterior flanks between tympanic region and posterior margin of axilla smaller than adjacent dorsal scales; MBSR 32; PRVSR 74; VS 69; enlarged preanal scales two, median scales overlapping outer; subcaudal scales 111, anterior in three rows, reducing to two at quarter of tail length and one row about half way to tail tip, slightly larger than surrounding scales. Trunk relatively long, TrunkL 29.7 mm, little more than half of SVL, more than addition of FIL and HIL, ratio of TrunkL/(FIL+HIL) 1.1; forelimb short, FIL 10.8 mm (FIL/SVL 0.21); forearm short, rather slender, FoL 7.4 mm (FoL/SVL 0.14); hindlimb longer than forelimb, HIL 16.6 mm (HIL/SVL 0.32), limbs separated by 2.3 mm when adpressed (4.4% of SVL); digits slender; SDLF4 11; SDLT4 16. 

**Coloration in life:** In life, the female holotype CBC02546 had the same color as female paratype CBC02840 (Figure 4). Dorsal surface of head, dorsum, and base of tail dark bronze-brown, side of head between tip of snout and forelimb insertion dark brown; dorsal surface of remaining tail reddish-brown. Dark broken regular dorsal stripes anteriorly and on tail (5) and posteriorly on body (7), formed by series of dark dots or elongated black spots, including wider dark paravertebral stripe; anterior part of dorsum with dorsal stripes formed by series of dots, posterior part of dorsum with dark dot-formed regular dorsal stripes reaching base of tail, continuing with dark stripes on dorsal surface of tail, extending about one-third of tail length; light laterodorsal stripes from behind eye, through temporals, along dorsolateral scale row to lateral sides of tail, and fading at one-third of tail length; large distinct regular black longitudinal dorsolateral stripe on each side of body, covering two to three scale rows, starting as narrow stripe covering about one scale row, running from posterior corner of eye through upper temporals, above tympanum, expanding wider to two scale rows above axilla, running below light dorsolateral stripe, along upper flanks through upper angle of groin to lateral surface of tail, becoming indistinct at posterior lateral tail (∼12 mm from tail tip); body flanks ventrally with whitish-beige longitudinal streaks and dark markings on bluish-brown background; ventrolateral surfaces from below level of eye to axillary region with longitudinal whitish-grey streaks and dark marking on reddish-brown background; lateral surfaces of tail reddish-brown; dorsal surfaces of limbs with irregular dark blotches on dark brown background. Ventral surfaces of head, gular region, body, and limbs uniformly white; ventral surface of tail uniform pinkish-cream. Palmar surfaces of hands and thenar surfaces of feet dark grey. Iris light-grey. 

**Coloration in preservative:** In preservative, dorsal surfaces of holotype turned dark greyish-brown; wide dorsolateral black stripe remained distinct; head with faint irregular dark spots, pineal ocellus present as single white dot on posterior part of interparietal; sides of head and supralabials with dark mottling; infralabials with dark spots; lateral sides of tail with small dark spots; throat and ventral surface of body and limbs greyish-cream; ventral surface of tail lighter cream; palmar surface of hand, fingers, and toes dark grey (Figure 3, Figure 7D, E, F). 

**Variation:** Paratypes (Table 1) resemble holotype in most morphometric and meristic characters and coloration. Noteworthy variation is that the holotype has six SL on each side of the head, whereas all paratypes have seven. CBC02545 has two supraoculars on left side, second posterior one larger with clear short suture indicating incomplete fusion of two posterior supraoculars; three supraoculars on right side, third with clear short suture indicating incomplete fusion with fourth posterior-most supraocular; one distinct pair of enlarged nuchal scales (remaining specimens have weakly enlarged nuchals) and first pair of chinshields in narrow contact with each other (vs. in broad contact in other specimens). CBC02545 has four postsuboculars and CBC02840 has five postsuboculars on both sides. Male paratype exhibits greater tail length (97.3 mm) than female holotype and paratypes. All specimens have six nuchal scales posterior to parietals, except subadult CBC02842, which has seven. In life, male shows distinctly more reddish-brown coloration of dorsum, with indistinct mid-dorsal stripe; throat, lower body flanks, ventral surfaces of body, lateral and ventral surfaces of tail in male show distinct reddish-orange coloration (Figure 4B). In preservative, reddish-orange coloration faded and became paler (Figure 7D). 

**Natural history:** The species was recorded from semi-deciduous lowland forests at elevations ca. 400–500 m a.s.l. in Keo Seima Wildlife Sanctuary from the eastern plain of Cambodia. Most specimens were encountered during the day, but juveniles CBC02843–45 were encountered at night among leaf litter. The holotype female CBC02546, paratype male CBC02545, and subadult CBC02841 were spotted moving near rotten logs, female CBC02840 was moving along the ground on the forest floor, and subadult CBC02842 was found under a rotten log. Diet and reproductive biology of the new species remain unknown. The gravid female holotype carried two eggs.

**Etymology:** The specific epithet is from the Latin words “*niger*” for “black” and “*fascia*” for “band”, in reference to the wide black dorsolateral stripes typical for this species. 

**Distribution:** To date known only from the type locality in Keo Seima Wildlife Sanctuary, O’Raing District, Mondulkiri Province, Cambodia at elevations ca. 400–500 m a.s.l.. However, the discovery of this species in adjacent areas of southern Vietnam is highly expected. 

**Comparisons:** The morphological characters distinguishing the new species from its Southeast Asian congeners are summarized in Table 4. Morphological comparisons of *Scincella* species found in Cambodia are given in Table 5. *Scincella nigrofasciata* **sp. nov.** can be diagnosed from *S. apraefrontalis* Nguyen, Nguyen, Böhme and Ziegler, 2010 of Vietnam by its longer SVL (40.0–52.6 vs. 36.1 mm), greater number of IL (6 vs. 5), DBR (8 vs. 4), MBSR (32–33 vs. 18), PRVSR (69–74 vs. 52), and VS (65–69 vs. 50), and prefrontals in broad contact (vs. prefrontals absent); from *S. monticola* (Schmidt, 1925) of Vietnam and China by having a longer SVL (40.0–52.6 vs. 31.8 mm), two primary temporals (vs. one), fewer EnLN (0–1 vs. 3–4), and greater number of DBR (8 vs. 4), MBSR (32 vs. 22–26), PRVSR (69–74 vs. 52–59), VS (65–69 vs. 52–58), and SDLT4 (15–17 vs. 10–13); and from *S. punctatolineata* (Boulenger, 1893) of Thailand and Myanmar by longer SVL (40.0–52.6 mm for three adults and single subadult specimen, SVL 50.2–52.6 mm for three adults vs. 37.6–40.2 mm), greater number of MBSR (32–33 vs. 22–28), two primary temporals (vs. one), and greater number SDLT4 (15–17 vs. 13–15). *Scincella nigrofasciata* **sp. nov.** can be distinguished from *S. darevskii* Nguyen, Ananjeva, Orlov, Rybaltovsky and Böhme, 2010 of Vietnam by having a much shorter SVL (40.0–52.6 vs. 88.6 mm), fewer supraoculars (2–4 vs. 5), two primary temporals (vs.one), and greater number of DBR (8 vs. 6), MBSR (32–33 vs. 28), and PRVSR (69–74 vs. 62); from *S. doriae* (Boulenger, 1887a) of Myanmar and China by having a shorter SVL (40.0–52.6 vs. 58.6 mm), fewer EnLN (0–1 vs. 3–5), slightly fewer VS (65–69 vs. 70–79), 5–7 discontinuous regular dark dorsal stripes (vs. dorsum caramel brown with small brown spots), and distinct wide black dorsolateral stripes (vs. dark brown dorsolateral stripes broken up by whitish spots); from *S. rara* Darevsky & Orlov, 1997 of central Vietnam by having fewer EnLN (0–1 vs. 3), greater number of MBSR (32–33 vs. 24) and PRVSR (69–74 vs. 53), and single row of basal subdigital pads (vs. double row of basal subdigital pads); from *S. victoriana* (Shreve, 1940) of Myanmar by having a shorter SVL (40.0–52.6 vs. 57.5 mm), fewer EnLN (0–1 vs. 3), smooth dorsal scales (vs. keeled), and a greater number of PRVSR (69–74 vs. 50–54) and VS (65–69 vs. 53–56). The new species can be distinguished from *S. ochracea* (Bourret, 1937) of Vietnam and Laos by its longer SVL in males (50.2 mm, *n*=1 vs. 34.2–45.4 mm, *n*=6), lack of lobules around external ear opening (vs. 2–4 lobules), dark brown dorsum with 5–7 discontinuous regular dark dorsal stripes (vs. silver-grey with a dark vertebral stripe), and distinct wide black dorsolateral stripes (vs. dark brown flanks broken up by light spots).

Among the Cambodian species, *Scincella nigrofasciata* **sp. nov.** can be distinguished from *S. melanosticta* (Boulenger, 1887b) of Cambodia, Myanmar, Thailand, and Vietnam by its comparatively shorter forelimbs (FIL/SVL 0.20–0.22 vs. 0.23–0.27), comparatively shorter hind limbs (HIL/SVL 0.30–0.33 vs. 0.35–0.37), adpressed limbs overlapping 0.4–2.2 mm in males and subadult specimens and separated by a 1.9–2.3 mm gap in adult females (vs. adpressed limbs widely overlapping 4.5–8.2 mm), two primary temporals (vs. one), fewer DBR (8 vs. 10), and 5–7 discontinuous regular dark dorsal stripes (vs. dark brown dorsum with dark dense spots without obvious striped pattern). The new species can be distinguished from *S. rupicola* by its comparatively shorter hind limbs (HIL/SVL 0.30–0.33 vs. 0.36–0.40), adpressed limbs overlapping 0.4–2.2 mm in male and subadult specimens and separated by a 1.9–2.3 mm gap in adult females (vs. adpressed limbs overlapping 3.5–7.2 mm), fewer SDLT4 (15–17 vs. 18–21), fully everted hemipenis bifurcating at 63% of total hemipenis length, *n*=1, Figure 5E (vs. 69%–77%, *n*=3, Figure 5F), comparatively more slender fingers and toes (Figure 5A vs. Figure 5B), 5–7 discontinuous regular dark dorsal stripes, Figure 4, Figure 7D–F (vs. dark blotches on dorsum in females and uniform reddish brown pattern without dark markings in males in *S. rupicola*; Figure 6A–B, Figure 7B–C).

In both morphometric and meristic characters *Scincella nigrofasciata* **sp. nov.** is most similar to *S. reevesii* and *S. rufocaudata*. However, the new species can be distinguished from *S. reevesii* by slightly shorter forelimbs (FIL/SVL 0.20–0.22 vs. 0.24–0.30), generally shorter hind limbs (HIL/SVL 0.30–0.33 vs. 0.34–0.43), comparatively shorter forearms (FoL/SVL 0.14–0.16 vs. 0.17–0.19, Table 5), adpressed limbs overlapping 0.4–2.2 mm in males and subadult specimens and separated by a distance of 1.9–2.3 mm in females (vs. overlapping 3.9–6.5 mm in both sexes), 5–7 dark discontinuous regular dark dorsal stripes (vs. irregular dark vertebral line and dark dorsal spots), wide distinct black dorsolateral stripes, continuing to lateral sides of tail, Figure 4 (vs. dark dorsolateral stripes less distinct and broken up by light spots and only extending to tail base in both sexes, Figure 6E, D), comparatively more slender fingers and toes (Figure 5A vs. Figure 5C), and dark brown palmar surfaces of hands and lower surface of fingers and toes, Figure 5A (vs. light grey palmar surfaces of hands, fingers and toes, Figure 5C).

*Scincella nigrofasciata* **sp. nov.** can be distinguished from *S. rufocaudata* by prefrontals in broad contact (vs. prefrontals separated), comparatively shorter hind limbs, (HIL/SVL 0.30–0.33 vs. 0.37), fewer IL (6 vs. 7), fewer DBR (8 vs. 10, Table 4, Table 5, Table 6), 5–7 discontinuous regular dark dorsal stripes (vs. 1–3 dark stripes with spots, Figure 8), and distinct wide black dorsolateral stripes continuing along tail (vs. black stripes broken, ending at tail base, Figure 4) (Table 5).

## DISCUSSION

Our work clearly demonstrated the new species from Mondulkiri Province to be distinct from other *Scincella* species known from Cambodia, including *S. melanosticta*, *S. reevesii*, *S.* cf. *rufocaudata, and S. rupicola*. Data on their morphological differences are summarized in Table 4, Table 5 and Figure 5, Figure 6 and Figure 7. As the original description of *Sphenomorphus rufocaudatus* by Darevsky & Nguyen (1983) was quite short and published only in Russian, we provided additional morphological information (Table 5 and Table 6) and photos (Figure 8) of the holotype ZISP 19797.

To facilitate future work on the genus *Scincella* of Cambodia, we provide the following comparisons between the named species, based on specimen examination and character states taken from the literature (Table 4). *Scincella melanosticta* can be distinguished from *S. reevesii* by one primary temporal (vs. two) and greater number of DBR (10 vs. 8). Both male and female *S. melanosticta* have a dorsum with dense dark spots (dark spots on almost every dorsal scale) and lack conspicuous dorsal stripes (vs. vertebral/irregular lines of dorsal spots in *S. reevesii*, except some male individuals of *S. reevesii*, probably in the breeding season, show a reddish brown dorsum that lacks dorsal spots), and distinct dark dorsolateral stripes interrupted by irregular light transverse bars/spots (vs. dark dorsolateral stripes, running along upper flanks and less interrupted by light spots). The bifurcated part of the hemipenis is 56% its total length (*n*=1) in *S. melanosticta* (vs. 65%–68%, *n*=2, in *S. reevesii*).

*Scincella melanosticta* can be distinguished from *S. rufocaudata* by prefrontals in contact (vs. prefrontals separated), one primary temporal (vs. two), longer forearms (FoL/SVL ratio 0.17–0.20 vs. 0.15), SDLT4 21–22 (vs. 15), dorsal dark brown with dense pattern of dark spots (vs. ochre with 1–3 dark stripes and spots), and dark dorsolateral stripes interrupted by irregular light transverse bars/spots in both sexes (vs. distinct, regular dark dorsolateral stripes (Figure 6 and Figure 7 for *S. melanosticta*).

*Scincella melanosticta* can be differentiated from *S. rupicola* by having one primary temporal (vs. two), greater number of DBR (10 vs. 8), dorsum with dense dark dorsal spots in both sexes (vs. dark blotches with pair of smaller blotches on neck in females only, whereas males lack blotches or have very faint dark dorsolateral stripes), and heavy dark spots on sides of head, extending above axillary region and below dorsolateral stripes along flanks in both sexes in *S. melanosticta* (vs. dark mottling in females and no dark markings in males in *S. rupicola* (Figure 6A-C; Figure 7A-C).

*Scincella reevesii* can be distinguished from *S. rupicola* by its higher TaL/SVL ratio 1.57–1.73, *n*=3 (vs. 1.06–1.53, *n*=5), dark brown dorsum with small vertebral/irregular dorsal spots (vs. dorsal blotches and pair of smaller blotches on neck only in females), distinct wide dark dorsolateral stripes, running along upper flanks, almost not interrupted by light spots (vs. large dark blotches interrupted by light bars in females), and body slender in males (vs. body in males comparatively thicker in *S. rupicola*) (Figure 6A, B, D, E; Figure 7G–H).

*Scincella rufocaudata* can be distinguished from *S. rupicola* by prefrontals separated (vs. in broad contact), shorter forelimbs (FlL/SVL 0.22 vs. 0.23–0.26), adpressed limbs overlapping at about 1.8 mm (vs. 3.5–7.2 mm), dorsal ochre with 1–3 dark brown spots (vs. dark brown dorsal pattern with blotches in females and without blotches in males), and slender body (vs. comparatively thicker in *S. rupicola*). The dorsal pattern with dark blotches and a few pairs of smaller blotches on neck in females and characters stated in Table 4 match the diagnosis of *S. rupicola* by Taylor (1963). *Scincella rupicola* has been reported from Thailand, Laos, and Vietnam (Nguyen et al., 2010b; Taylor, 1963; Teynié et al., 2004), but has not been reported previously from Cambodia. Herein we identify this species as *S.* cf. *rupicola* for the first time from Cambodia. This species ranges from the central part, Kampong Thom (Hayes et al., 2015) to Siem Reap Province (T. Hartmann, pers. comm.).

Morphologically, *S. reevesii* is superficially similar to *S. rufocaudata* (Table 4 and Table 5). It can be distinguished from *S. rufocaudata* by its prefrontals in broad contact (vs. separated), adpressed limbs more widely overlapping (3.9–6.5 vs. 1.8 mm), upper secondary temporal enlarged (vs. not enlarged), and DBR 8 (vs. 10); see Table 5 and Table 6 for more detail.

We examined photographs of specimens (FMNH 263355–58) from the Cardamom Mountains of southwest Cambodia, deposited at the Field Museum of Natural History, which were assigned to *S. rufocaudata* by Stuart & Emmett (2006), and suggest that these specimens appear more like *S. reevesii* than *S. rufocaudata* based on their dark dorsolateral stripes, which are more continuous and interrupted by less distinct light spots/bars. Two (CBC01380 and CBC02305) out of seven male specimens from the Cardamom Mountains of southwest Cambodia that we assigned to *S. reevesii* have prefrontals slightly separated and another male (CBC01379) has prefrontals in narrow contact, which we suggest is a variation within this population of *S. reevesii*.

We examined photographs of specimens (FMNH 262998–99) collected 3–4 km from the new species type locality in Mondulkiri on the Cambodian eastern plain, which were assigned to *S. rufocaudata* by Stuart et al. (2006). These specimens look different from *S. rufocaudata*, based on their prefrontals in contact as opposed to prefrontals separated in *S. rufocaudata*; and differ from *S. reevesii* in having more distinct wide dark dorsolateral stripes; they also differ from the new species based on their light brown dorsal coloration, and lower edge of dorsolateral stripes more interrupted by irregular light spots. However, because these specimens were inaccessible to us, we suggest retaining the specimens FMNH262997–3001 reported by Stuart et al. (2006) from Mondulkiri as *Scincella* cf. *rufocaudata*, pending further studies and genetic analyses.

The discovery of a new species of *Scincella* in Mondulkiri Province brings the named species of *Scincella* known for Cambodia to five, namely, *Scincella nigrofasciata* **sp. nov.**, *S. melanosticta*, *S. reevesii*, *S.* cf. *rufocaudata*, and *S. cf. rupicola*, adding another species of lizard to the country. The new species seems to be morphologically quite variable; therefore, additional adult specimens and further studies are required to assess morphometric and meristic character variations.

To date, *Scincella nigrofasciata* **sp. nov.** is known only from the type locality in the Mondulkiri Province of Cambodia. However, it is very likely that the new species inhabits other hilly areas of the southern outcrops of the Annamite Mountains in adjacent areas of Vietnam (e.g., Binh Phuoc, Lam Dong, and Dong Nai Provinces), and thus further morphological and molecular studies are needed to confirm the extent of its distribution in southern Indochina. The discovery of a new species of *Scincella* indicates that the reptile fauna of Keo Seima Wildlife Sanctuary remains insufficiently studied and future field surveys are needed to assess its herpetodiversity. This study further highlights the importance of taxonomic research and biodiversity assessments for nature conservation.

## APPENDIX I

Examined materials:

*Scincella nigrofasciata* **sp. nov.** (8 specimens): CBC02545–46, CBC02840–45: O’Raing District, Mondulkiri Province, eastern plain, Cambodia.

*Scincella melanosticta* (8 specimens): CBC01009, CBC01020–22, CBC01430, CBC01434, CBC01450, CBC01454: Phnom Samkos Wildlife Sanctuary, Veal Veng District, Pursat Province, Cardamom Mountains, Cambodia.

*Scincella reevesii* (9 specimens): CBC01357–58, CBC01149, CBC01342, CBC01379–80, CBC01382, CBC01479, CBC02305: Phnom Samkos Wildlife Sanctuary, Cardamom Mountains, southwest Cambodia.

*Scincella rupicola* (10 specimens): CBC02407–10, CBC02412–15: Phnom Chi, Sandan District, Kampong Thom Province, Prey Lang; CBC02323, CBC02339: Karst, Thalavorivath District, Stung Treng Province, northern Prey Lang.

*Scincella rufocaudata*: holotype of *Sphenomorphus rufocaudatus* (Darevsky & Nguyen, 1983): ZISP 19797 Coll. Darevsky I.S. 16–21.06.1983, Buon Luoi, Kon Tum – Gia Lai Province (now in Gia Lai Province), Vietnam.
